# Developing self-efficacy and ‘communities of practice’ between community and institutional partners to prevent suicide and increase mental health in under-resourced communities: expanding the research constructs for upstream prevention

**DOI:** 10.1186/s12889-025-22465-1

**Published:** 2025-04-08

**Authors:** Lisa Wexler, Lauren White, Joel Ginn, Tara Schmidt, Suzanne Rataj, Caroline C. Wells, Katie Schultz, Eleni A. Kapoulea, Diane McEachern, Patrick Habecker, Holly Laws

**Affiliations:** 1 Department of Social Work and Research Center for Group Dynamics, Institute for Social Research, University of Michigan, Ann Arbor, MI 48104 USA; 2 School of Social Work, University of Washington, 4101 15th Ave NE, Seattle, WA 98105 USA; 3 Department of Psychology and Neuroscience, Boston College, McGuinn 300, 140 Commonwealth Ave, Chestnut Hill, MA 02467 USA; 4 Department of Health Promotion and Policy, University of Massachusetts, Amherst, 01003 USA; 5 Division of Social and Transcultural Psychiatry, McGill University, Montreal, H3A 0G4 Canada; 6 Center for Research on Families and Psychological and Brain Sciences, University of Massachusetts, Amherst, 01003 USA; 7 Rural Drug Addiction Research Center, University of Nebraska-Lincoln, Lincoln, NE 68588 USA; 8 University of Alaska Fairbanks, Kuskokwim Campus, Bethel, AK 99559 USA

**Keywords:** Suicide prevention, Health promotion, Communities of practice, American Indian/Alaska native, Rural, Wellness

## Abstract

**Background:**

Suicide is a serious and growing health inequity for Alaska Native (AN) youth (ages 15–24), who experience suicide rates significantly higher than the general U.S. youth population. In under-served, remote AN communities, building on existing local and cultural resources can increase uptake of prevention behaviors like lethal means reduction, interpersonal support, and postvention by family members, workers and community members, which can be important for preventing suicide in places where mental health services are sparce. This study expands the variables we hypothesize as important for reducing suicide risk and supporting mental wellness. These variables are: (1) perceived *suicide prevention* self-efficacy, (2) perceived *wellness* self-efficacy, and (3) developing a ‘community of practice’ (CoP) for prevention/wellness work.

**Method:**

With a convenience sample (*N* = 398) of participants (ages 15+) in five remote AN communities, this study characterizes respondents’ social roles: institutional role if they have a job that includes suicide prevention (e.g. teachers, community health workers) and community role if their primary role is based on family or community positioning (e.g. Elder, parent). The cross-sectional analysis then explores the relationship between respondents’ wellness and prevention self-efficacy and CoP as predictors of their self-reported suicide prevention and wellness promotion behaviors: (1) working together with others (e.g. community initiatives), (2) offering interpersonal support to someone (3), reducing access to lethal means, and (4) reducing suicide risk for others after a suicide death in the community.

**Results:**

Community and institutional roles are vital, and analyses detected distinct patterns linking our dependent variables to different preventative behaviors. Findings associated wellness self-efficacy and CoP (but not prevention self-efficacy) with “working together” behaviors, wellness and prevention self-efficacy (but not CoP) with interpersonal supportive behaviors; both prevention self-efficacy and CoP with higher postvention behaviors. Only prevention self-efficacy was associated with lethal means reduction.

**Conclusions:**

The study widens the scope of suicide prevention. Promising approaches to suicide prevention in rural low-resourced communities include: (1) engaging people in community and institutional roles (2), developing communities of practice for suicide prevention among different sectors of a community, and (3) broadening the scope of suicide prevention to include wellness promotion as well as suicide prevention.

Suicide is a leading cause of death for young people ages 10 through 24 in the United States [1, 2]. Preventive efforts are especially urgent for American Indian and Alaska Native (AIAN) youth whose elevated rates [1, 3] have increased significantly in recent years [4]. Similarly, rates of suicide in rural areas have increased 1.5 times faster than in urban areas in the United States from 1981 to 2018 [1]. Although the definition of rurality has been critiqued for being overbroad [5], generally, the association between suicide and rural America has been linked to economic distress and access to care [6]. More specifically, suicide in rural areas is linked to limited access to mental healthcare generally, mental health provider shortages, and stigma around mental health help-seeking [7, 8]. For AIAN young people, these factors are likely exacerbated by culturally incongruous care [9]. The cultural differences between therapists and the communities they serve can reduce the acceptability and impact of mental health services particularly to prevent suicide for AIAN people [10, 11]. For communities with histories of systemic injustice, oppressive practices such as involuntary inpatient treatment that is linked to suicide interventions, contribute to many not seeking mental health services [12]. These issues are particularly relevant in rural locations and in racially and ethnically marginalized communities [13–16], and in the remote, roadless predominately AN region of this study, particularly acute [11].

With suicide rates highest in rural areas and fastest growing among younger people of color [17–19] where culturally-responsive mental health services are sparce, there is an urgent need to understand the factors that can contribute to the success of a broad public health approach to suicide prevention. Such an approach includes more than professional mental health services, and builds on the more readily-available community, cultural and social resources in rural and diverse communities (e.g., Elders, parents, family members, mentors) to address suicide [20–23]. A public health approach to suicide prevention engages diverse collaborators within different community sectors (e.g., law enforcement, schools, religious organizations) and within young people’s existing social support networks [24–26]. Such multilevel efforts can include offering interpersonal support to young people, bolstering family support systems, and developing community-level opportunities that promote youth wellbeing and reduce suicide risk. Previous research suggests that such initiatives can be leveraged to create community-based, culturally- and locally-appropriate strategies [21, 23, 27].

A public health approach to suicide prevention targets multiple levels of influence on health and mental health across societal, community, family and individual levels [28]. These strategies include universal, selective and indicated spheres of prevention [28] and require multisector cooperation to support people– especially young people with intersecting marginalized identities– in the settings they frequent such as schools, religious and sport organizations, homes (parents and extended family members) [29]. Indigenous theories of holistic health align well with this framework; for example [30], found that cultural continuity—maintaining community values, practices, and social support systems—serves as a protective factor against youth suicide. Further, Indigenous resilience emphasizes that wellness arises from social relationships, collective identities, and traditional practices [31, 32] and community-engaged research across many different AIAN populations has often yielded strengths-based, multi-level, and upstream suicide prevention initiatives [33]. Building from the intersecting lenses of public health and AIAN research and theory, this study highlights two significant, often overlooked, aspects of universal suicide prevention: building Communities of Practice (CoP) that include people in both community and institutional support roles, and self-efficacy to take upstream action for suicide prevention, including actions for mental health wellness promotion as well as prevention or reduction of suicide risk.

## Communities of practice

Developing collaborative relationships for suicide prevention and wellness, also described as “communities of practice” (CoP) [34] can be important for locally-driven and adaptive prevention strategies [35] as well as sustainment of suicide prevention strategies, particularly in under-resourced communities. Rooted in social learning theory, CoP are groups of people that cultivate three inter-related elements: shared interest, common community, and practice [34]. Separate from institutional or organizationally based groups, CoPs are defined by a collective process of engaging, sharing information, and doing [36]. Often, people within a CoP represent different perspectives and areas of expertise and therefore learn about and address complex issues from multiple angles [37]. Since the effects of colonization have unfolded at multiple levels, eroding the agency of AIAN communities by disrupting traditional governance and social support structures [38, 39], leveraging CoPs to build community practice across community and institutionally oriented social groups is fitting. CoP can strengthen AN community helping networks by linking across community structures and institutional services and systems. This can help demystify and destigmatize mental health and wellness, and foster increased interactions between local helpers (e.g., parents, Elders) and institutional helpers., (service providers, teachers), making it easier for integrated and coordinated care to occur when a young person is struggling but not yet actively suicidal [40]. Through engagements in CoP, institutional helpers can connect with communities and families they serve, and get feedback for practicing cultural humility, making their services more locally responsive [41]. Social networks of support are an important and often overlooked resource to engage in suicide prevention, particularly in under-resourced communities where institutional services are limited [42]. Building CoP [34, 37] that include AN community and family resources (i.e. community leaders, parents, etc.) as well as people in institutional or professional roles (i.e. community health workers, teachers, social workers) is an innovative way to address the complex and culturally-specific issue of youth suicide prevention.

### Wellness and prevention self-efficacy

Our measured behavioral suicide prevention constructs test whether respondents in institutional or community/family roles endorsed participation in “promotion behaviors” (actions done to attain a positive outcome, for example: “I talked with someone about how culture can promote youth wellness”) and/or in “prevention behaviors” (actions done to avoid a negative outcome, for example: “I helped someone who was down get help”). This distinction between “prevention” and “promotion”-oriented behaviors aligns with Regulatory Focus Theory, which posits that people are motivated to pursue goals from these two different orientations, depending on personality and circumstances of an action [43, 44]. Promotion behaviors for health can generally be done regularly, regardless of circumstances within family and community life. Emergent opportunities to do “prevention” behaviors occur less frequently in daily life because they are predicated on risk detection or observed struggles. Thus, for people in community and family roles such as parents, teachers, coaches, Elders, these behaviors are more challenging to capture over a period of a few months. It is important to include both prevention and promotion in suicide prevention efforts.

**Fig. 1:**
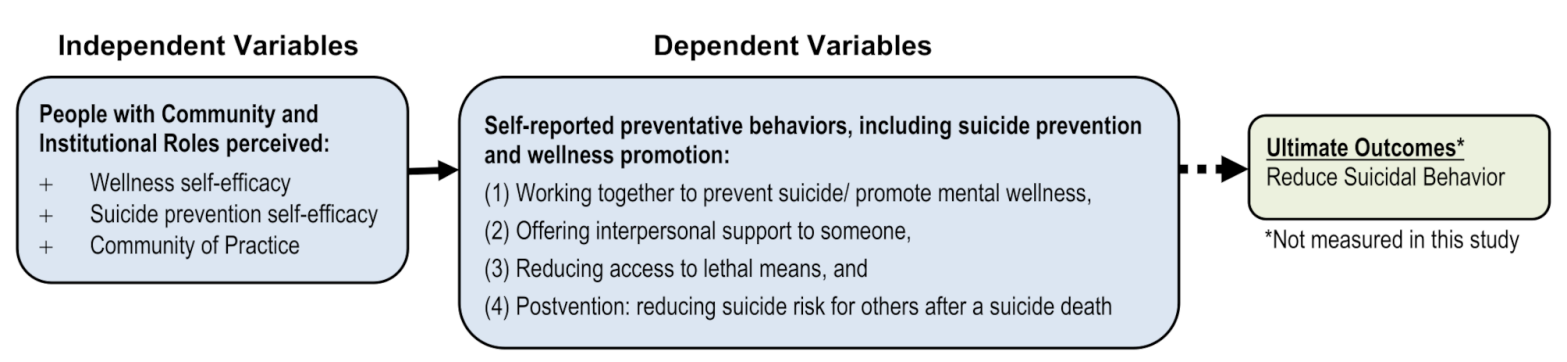
Theoretical model of study constructs

Our study assesses how *wellness* self-efficacy and *suicide prevention* self-efficacy relate to self-reported preventative behaviors of community members and considers participants’ collaborative relationships or CoP. Put differently, our theoretical model includes those in community and institutional roles and asks about their social relationships (i.e. CoP) that both enable supportive interactions with people who are suffering and offer resources and support to enhance and sustain culturally-responsive and culturally-based practices to enhance mental wellbeing and to intervene when someone is struggling to prevent suicidal behavior (see Fig. 1).

Our cross-sectional survey research from five Alaska Native (AN) rural and remote communities offer a snapshot of community members’ self-perceptions and self-reported behaviors–including suicide prevention and health promotion hereafter referred to as “preventative behaviors”—to assess the relationships between these key factors. Our analysis considers the preventative behaviors of people in different kinds of roles within community (family, friends, and Elders) and institutional (behavioral health and mental health counselors, teachers, community health workers) support networks of young people. We have updated our use of the terms “community” instead of “informal” and “institutional” in lieu of “formal” to acknowledge the important ways in which cultural and local protocols structure social relationships, and to reflect language that does not marginalize these social structures of engagement. Our study highlights how survey respondents’ self-perceptions and current collaborations support a variety of preventative behaviors. Our measures of prevention behaviors include a broad range of activities done with others (“Working together to prevent suicide and promote health”), offering interpersonal support, reducing access to lethal means (i.e. firearms) and reducing risk for others after a suicide death in the community (i.e. postvention). Suicide prevention requires a breadth of activities [18–24, 28] as well as collaboration across and among these social networks may be important for sustaining suicide prevention and wellness promotion activities, particularly in tight-knit communities. To date, little research considers the role of collaborative relationships in diversifying approaches to suicide prevention to include broad prevention strategies, including wellness promotion, that can be put into practice across multiple community sectors [25, 45].

## Methods

### Participatory approach

This analysis grows from over 25 years of partnership with AN communities focused on participatory research for suicide prevention. This study was conducted in collaboration with regional tribal health and social services organizations and local tribes where the research took place. In keeping with the principles and practices of community-based participatory research methods, the study design and measures were informed by a Local Steering Committee and the research was done under their guidance to benefit local communities as well as to advance the science. The PC CARES steering committee meets on a monthly basis, and members are paid for contributing their guidance and oversight at each meeting. More details about our participatory practice can be found in other PC CARES papers [46–51].

### Key constructs

We describe the connections between the self-perceptions of people in community (family, friends, and Elders) and institutional (counselors, teachers, community health workers) roles, which includes their self-efficacy related to both wellness (“There are things I can do to promote wellness here.”) and suicide prevention (“I feel confident that I can do things to prevent suicide.”). Our analyses describe the relationship of these self-perceptions and CoP constructs (i.e., “I have many people to work with in my community to prevent suicide.”) to participants’ self-reported preventative behaviors, which includes a wide range of health and mental health promotive behaviors as well as those aimed at suicide prevention.

These preventative behaviors reflect actions at the community, family and interpersonal levels. These include community-level actions such as *“working together”* to support youth wellness and prevention suicide (e.g., working with a group of people in the community to educate others, such as “I spoke up about what community organizations can do to reduce the risk of youth suicide.”); offering *“interpersonal support”* (e.g. encouraging help-seeking for someone who is down, such as “I reached out to someone who was hurting (alone, sad, angry).”); *“reducing access to lethal means”* (e.g., “I discussed how to make a home safer (no alcohol, gun safes).”); and *“postvention”* which involves precautions to reduce the risk of contagion if a suicide should occur (e.g., “I shared that it can be harmful to honor someone who died by suicide more than is done for other deaths.”) These categories of behaviors include a range of prevention strategies and are intentionally broad so can be enacted by a variety of people within a young person’s institutional and community social network. Notably, our dependent variables include actions taken within families and communities for both universal health promotion (i.e., to support wellbeing) as well as suicide prevention (i.e., done in response to risk) as important behavioral outcomes.

### Recruitment procedures and sample

We used baseline survey data collected as part of the Promoting Community Conversations About Research to End Suicide (PC CARES) initiative [see Wexler [40, 47] or www.pc-cares.org for more information on PC CARES]. These data were collected in fall 2019 from residents in five communities in rural, remote Alaska. The 20-minute electronic survey was completed on electronic tablet (iPads) and included questions about self-perceptions of suicide prevention and wellness self-efficacy, CoP, and preventative actions taken “within the past few months” both for promoting general wellbeing and buffering risk at times of struggle (suicide prevention via lethal means reduction and postvention, wellness, and supportive interpersonal interactions).

Participants were recruited by posting informational flyers in high traffic areas around each community, and by word of mouth. Participants were compensated twenty dollars in cash for completing the survey. Surveys were administered over a period of 1–2 days at central locations in each village (e.g., churches, schools, tribal buildings) by a PC CARES coordinator (*BLINDED*) who grew up in the participating region. Across the five communities (population ranging from 193 to 975 inhabitants), 430 people filled out surveys (about 15% of the pooled population across participating villages) with more people recruited in bigger villages.

### Steps to prevention survey development

We worked closely with the PC CARES Local Steering Committee (LSC), made up of people who live and work in the rural communities where data collection took place, to develop and adapt our survey. Starting with survey items that showed positive change in the pilot research for PC CARES pilot project between 2014 and 2016 [47], we worked with our LSC to collaboratively develop new questions and adjust survey items to ensure they fit with changes in curriculum, were clear and communicated the intended meaning [49]. The adapted “Steps toward Prevention Survey” (StP) was piloted with 100 people in the region to assess understandability, ease of use and preliminary psychometrics. See Table 1 for all items and associated reliability of each subscale.

**Table 1:** PC CARES steps to prevention (StP) measure

Construct Name	Item Text	Cronbach’s α	
**PREDICTORS**			
**Suicide Prevention Self Efficacy**			
	I know how to talk safely about suicide, in ways that help with prevention		**α = 0.779**
	I know how to decrease suicide risk for others by the way I talk about suicide		
	I know how to support someone who is at risk, whether or not I am close to them		
	I feel confident that I can do things to prevent suicide		
**Wellness Self Efficacy**			
	There are things I can do to promote wellness here		**α = 0.842**
	I know how to create a healthy environment for youth as they grow up		
	I know how I can make positive changes for community wellness		
**Community of Practice**			
	Many people in this community work together for suicide prevention/wellness		**α = 0.727**
	I have regular opportunities to work with others to increase wellness		
	I have many people to work with in my community to prevent suicide		
**BEHAVIORS**			
**Work Together to Prevent Suicide and Promote Health**			
	I asked someone for help doing prevention/wellness work when I needed it		
	I spoke up about what community organizations can do to reduce the risk of youth suicide		**α = 0.841**
	I suggested ways community organizations could work together to increase wellness		
	I talked with community members about wellness		
	I talked with others about wellness and/or suicide prevention		
	I worked with others to prevent suicide or promote wellness		
	I let people know what resources are available for prevention		
	I worked with a group of people in the community to share suicide prevention information		
**Interpersonal Support**			
	I spent time listening to someone who just wanted to talk about their experience		
	I trusted others in the community to hear what I have to say		**α = 0.689**
	I reached out to someone who was hurting (alone, sad, angry)		
	I helped someone who was down get help (Behavioral Health Services, Alaska Careline, etc.)		
	I reminded someone that just listening to someone can be more supportive than giving advice		
	I quietly listened to someone who had a problem, reflecting back to them what I heard.		
	I encouraged others to offer small acts of kindness when someone was having a hard time		
**Lethal Means Reduction**			
	I tried to make a home safer (such as no alcohol, locked guns) when worried about someone living there.		**α = 0.498**
	I worked on a community-wide project to make homes safer		
	I discussed how to make a home safer (no alcohol, gun safes)		
**Postvention**			
	I shared only the basic facts of a suicide (avoiding details)		
	I spoke to someone about how to talk safely after a suicide		**α = 0.742**
	I talked about how suicide is no one’s fault		
	I shared that it can be harmful to honor someone who died by suicide more than is done for other deaths		
	I talked about how we can help prevent further harm after a suicide happens.		

Our independent variables are self-reported behaviors: wellness self-efficacy, prevention self-efficacy, and CoP, and were rated on a Likert scale ranging from Strongly Disagree = 1 to Strongly Agree = 7. Each factor composite was calculated as an item average. The StP Survey dependent variables focus on different kinds of suicide preventative activities and are characterized as: [1] *Working together to prevent suicide and promote health* (8 items which includes 2 wellness and 3 suicide prevention actions, and 3 actions encompassing both) [2], *Interpersonal support* (7 items, of which 4 focused on wellness promotion, and 3 on suicide prevention actions) [3], *Lethal means reduction* (3 items, all of which focus on prevention actions), and [4] *Postvention* (8 items, all of which focus on prevention actions). There are more promotion items overall, especially for working together and community of practice constructs. We ask respondents to report their behaviors as they relate to the above constructs “in the last few months.” Actions were rated as binary endorsements (1 = Yes, 0 = No) of a list of suicide prevention and promotion behaviors and summed to form behavior scales in each domain. This process resulted in our final survey (see Table 1 for items and Cronbach’s alpha for each subscale) [41].

Given our focus on developing a community of practice, our study explores the roles held by our participants and how people in various positions in the community perceive their own knowledge and confidence related to prevention and how they interact to do prevention activities. The role distinction was self-selected without exception. “High School student” was a role participants could choose on the survey, but for analysis purposes this category was included in the “no role” category.

### Data analysis

All analyses were conducted using IBM SPSS Statistics (version 28) software [52] for our final sample of *N* = 398.

#### Analyses

Our analysis considers the associations between self-perceptions, community of practice and participants’ preventative behaviors. We applied familywise test corrections using the Holm-Bonferroni sequential method to determine the relationship between our independent variables of wellness self-efficacy, suicide prevention self-efficacy and CoP and our dependent behavioral variables: working together, interpersonal support, lethal means reduction and postvention. To estimate the relationship between the self-perceptions and behaviors, each type of behavior was regressed on each self-perception construct to examine independent effects of each self-perception on behavior, while accounting for the effects of other self-perceptions. Using the role categories of participants, we conducted one-way ANOVAs and pairwise post-hoc tests to examine if there were differences in each of our measures of self-perceptions and actions between participants who held different types of roles in the community. The Holm-Bonferroni sequential method was used to correct for family-wise error for each association tested on the four behavioral outcomes, as behavior construct outcomes were correlated measures [53].

## Results

### Descriptive and exploratory analysis results

An analytic subset of *N* = 398 participants with complete data on all independent (predictor) and dependent (outcome) variables was used for all analyses. Of these 398 respondents, 182 were aged 15–29. We over-sampled young people since they represent the highest risk age group in Alaska [54, 55]. Our sample had the following role groupings: those with undefined roles and high school students (*n* = 120, 30%), community role only (*n* = 211, 53%), institutional role only (*n* = 25, 6%), or both institutional and community roles (*n* = 42, 11%). Descriptive statistics and bivariate Pearson correlations among study variables for each of the composites are provided in Table 2. Note that all variables were significantly and positively correlated with one another. For example, participants who rated their wellness self-efficacy higher also tended to endorse higher ratings of perceived self-efficacy around suicide prevention.

**Table 2:** Descriptive statistics and correlations for study independent and dependent variables

Variable	M	SD	1	2	3	4	5	6	7
1. Wellness self-efficacy	5.67	1.05	-						
2. Suicide Prevention self-efficacy	5.68	1.12	0.72	-					
3. Community of Practice	5.24	1.26	0.61	0.58	-				
4. Working Together to Prevent Suicide and Promote Health	3.14	2.62	0.53	0.43	0.50	-			
5. Interpersonal Support	5.20	1.69	0.43	0.41	0.34	0.54	-		
6. Lethal Means Reduction	1.75	0.91	0.35	0.40	0.31	0.50	0.40	-	
7. Postvention	2.86	1.92	0.46	0.54	0.43	0.70	0.61	0.51	-

All correlations were statistically significant, *p* <.001

**Table 3:** Role differences in study variables

		Participant Role								Omnibus Test of Group Differences		Pairwise Differences
		Other Role *n* = 120		Community Role *n* = 211		Institutional Role *n* = 25		Institutional & Community *n* = 42				
		M	SD	M	SD	M	SD	M	SD	F (df = 3,394) *p*		
IV: Self-Perceptions	Wellness Self Efficacy	5.43	1.06	5.70	1.02	6.00	0.94	6.01	1.12	4.43	0.004	_***a, b,c***_
	Suicide Prev. Self-Efficacy	5.52	1.14	5.72	1.12	6.07	1.12	5.68	1.07	1.96	0.308	_−−_
	Community of Practice	5.17	1.22	5.21	1.28	5.66	1.12	5.33	1.37	1.17	0.321	_−−_
DV: Wellness/ Prevention Behaviors	Working Together	2.69	2.48	3.17	2.63	4.08	2.90	3.71	2.65	2.96	0.032	_***b, c***_
	Interpersonal Support	4.93	1.83	5.23	1.60	5.92	1.68	5.38	1.65	2.78	0.041	_***c***_
	Lethal Means Reduction	1.54	0.93	1.85	0.84	1.84	1.11	1.76	0.96	3.15	0.025	_***a***_
	Postvention	2.64	2.01	2.96	1.87	3.32	1.81	2.69	1.92	1.32	0.266	_−−_

Note: ANOVA Significant pairwise differences reported only where F test was significant, and are indicated as:

***a*** Participants endorsing no role differ from those endorsing community roles only

***b*** Participants endorsing no role differ from those endorsing institutional roles only

***c*** Participants endorsing no role differ from those endorsing both institutional & community roles.

No significant pairwise differences were found for any other role comparison aside from the three outlined above.

**Table 4:** Associations between Self-Perceptions and suicide prevention and promotion behaviors

	*Working Together*			*Interpersonal Support*			*Lethal Means Reduction*			*Postvention*		
	B [95%CI]	SE	β	B [95%CI]	SE	β	B [95%CI]	SE	β	B [95%CI]	SE	β
Wellness Self Efficacy	**0.86** ^***^ **[0.55**,** 1.17]**	**0.16**	**0.34**	**0.40** ^***^ **[0.18**,** 0.62]**	**0.11**	**0.25**	0.09 [-0.03, 0.21]	0.06	0.10	0.16 [-0.07, 0.39]	0.12	0.09
Suicide Prevention Self-Efficacy	0.05 [-0.23, 0.34]	0.15	0.02	**0.28** ^**^ **[0.08**,** 0.48]**	**0.10**	**0.18**	**0.22** ^***^ **[0.11**,** 0.33]**	**0.06**	**0.27**	**0.66** ^***^ **[0.45**,** 0.87]**	**0.11**	**0.38**
Community of Practice	**0.57** ^***^ **[0.35**,** 0.79]**	**0.11**	**0.28**	0.12 [-0.04, 0.27]	0.08	0.09	0.06 [-0.03, 0.14]	0.04	0.08	**0.23** ^**^ [**0.06**,** 0.39]**	**0.08**	**0.15**
*Model R* ^2^	*R*^*2*^*=* 0.33 *F*(3,394) = 64.34^***^			*R*^*2*^*=* 0.21 *F*(3,394) = 35.05^***^			*R*^*2*^*=* 0.17 *F*(3,394) = 26.98^***^			*R*^*2*^*=* 0.31 *F*(3,394) = 58.74^***^		*R*^*2*^*=* 0.21 *F*(4,393) = 26.09^***^

*p <.05, **p <.01, ***p <.001. Bold coefficients indicate findings which held after applying familywise test corrections using the Holm-Bonferroni sequential method. The pattern of these results remains the same even after controlling for role differences

We examined differences in all study variables by participants’ social roles: institutional role (e.g. behavioral health and mental health counselors, teachers, community health workers) (*n* = 25, 6%), community role (e.g., parent, Elder) (*n* = 211, 53%), those who endorsed both institutional and community roles (e.g., both parent and community health worker, *n* = 42, 11%), and those who reported holding neither institutional or community roles (e.g. not currently employed, high school students, younger people not yet parents) (*n* = 120, 30%). Groups were compared using one-way ANOVAs, with follow-up pairwise comparisons performed with no familywise correction to explore potential role differences in our study variables.

For the self-perception independent variables, a significant group difference was found for wellness self-efficacy. Those endorsing institutional or community roles (or both) had significantly higher ratings of wellness self-efficacy than those not endorsing these roles. For the behavior composites, those endorsing a community and/or institutional role were more likely to work together with others to prevent suicide and promote health and to offer interpersonal support to others. Those with an undefined role (did not select one) were less likely to take action to reduce someone’s access to lethal means as compared with those with who selected a community and/or institutional role. Pairwise follow-up comparisons indicated that those endorsing no institutional or community roles reported engaging in significantly fewer prevention and wellness behaviors compared with other groups. The analysis found no other significant differences between roles, including tests to control for age and gender. Table 3 provides means on each subscale by role group and includes relevant statistics from the ANOVAs.

To test our hypotheses linking self-perceptions to self-reported behavior, we conducted a separate multiple linear regression for each behavioral outcome construct (Working Together, Interpersonal Support, Lethal Means Reduction, and Postvention) with self-perceptions (Wellness Self-Efficacy, Suicide Prevention Self-Efficacy, and CoP) as simultaneous predictors. Results indicated distinct patterns of association of self-perceptions to behavior for each behavioral outcome. Higher self-ratings of wellness self-efficacy, as well as having collaborative relationships for suicide prevention or a CoP each were significantly and positively associated with the Working Together behavioral construct. Both self-efficacy ratings (prevention and wellness self-efficacy) were significantly associated with Interpersonal Support behaviors. The only statistically significant association with Lethal Means Reduction behaviors was suicide prevention self-efficacy. Finally, both suicide prevention self-efficacy and CoP were significantly associated with Postvention behaviors. Taken together, self-perceptions of wellness self-efficacy, prevention self-efficacy and CoP explained a substantial proportion of the variability in suicide prevention and health promotion behaviors, with *R*^*2*^ values ranging from 0.17 to 0.32. Importantly, follow-up analyses controlling for role differences found no significant effects for role type on the outcomes after controlling for self-perceptions, and the pattern and strength of results for these associations was consistent with models reported here (results for models controlling for Role available upon request). See Table 4 for statistical results from all multiple regressions.

## Discussion

Our study clearly emphasizes the importance of community adult supporters in both institutional and community roles (i.e., people within families and communities outside of institutional roles as well as professionals) as a vital source for enacting suicide prevention and wellness promotion activities within under-resourced Alaska Native communities. This finding is supported by a previous social network analysis describing young AN people’s social support networks in remote Alaskan communities where they rely predominantly on family members and peers for support [20]. Developing a community of practice (CoP) to enact a variety of community and family-based suicide prevention and wellness initiatives is novel, and this study provides evidence for this approach. Adult family and community members as well as social service providers (including community health workers, teachers, coaches) are offering a variety of preventative interactions and social support to young people. Additionally, our study supports an expanded conception of self-efficacy that includes health promotion as well as suicide risk reduction behaviors (i.e., lethal means reduction, postvention). These points push the field toward a more expansive approach to suicide prevention and offers clear ways to measure these variables, which have important implications. Each will be discussed here.

Often, suicide prevention interventions in the United States ignore the community, cultural, and family assets that our data show as vital (i.e., those adult supporters endorsing community roles) and relegates people occupying supportive family and community roles (i.e., Elders, parents, aunts, etc.) to positions of ‘gatekeepers’ who identify and refer vulnerable young persons to mental health care [56, 57] rather than active partners in this work [28]. The youth suicide prevention field has devoted much time and attention to this gatekeeper model [26, 56] even though evidence of this strategy’s mitigating impact on youth suicidal events is sparce, mixed, and ultimately inconclusive [57–59]. Most importantly, the aim of gatekeeper approaches is to assess individual risk and refer to clinical services, which are not often available in under-resourced communities [7, 8] nor are they typically culturally-responsive for marginalized young people [9]. Therefore, while the popular use of gatekeeper training may contribute to suicide prevention, it is not enough on its own; particularly in marginalized, under-resourced, and rural communities where youth suicide is a growing concern. This study provides an expanded perspective and identifies measures for tracking community-based efforts and interactions with preventative potential.

Our data underscores the importance of systems-based approaches that strengthens the cultural, community, and family resources which are often more plentiful in rural settings than clinical mental health services or institutional resources. Such approaches empower rural communities to leverage their existing resources and local expertise to reach the people in their communities who need help the most [60]. People who identified as occupying both community and institutional roles were likely to work together with others to prevent suicide and promote health (Table 2), supporting the notion that both community and institutional systems are important vectors for multidimensional and collaborative suicide prevention actions [61]. This finding highlights the important, often ignored, cultural and community strengths within communities that can be important assets for promoting wellness and preventing suicide *before* a crisis. Partnering with community and family members–those who are already engaged in the lives of youth–offers a way to strengthen the social safety net that is currently in place in communities. Indeed, such collective efforts may support more sustainable and larger-scale outreach for suicide prevention and health promotion in rural, under-served communities, like the remote AN communities in this study.

Likewise, in our analysis, people who indicated having an existing CoP to work with (i.e., “I have regular opportunities to work with others to increase wellness”) were more likely to report taking actions in collaboration with others to address suicide (Table 3). These multidimensional activities included collaborative efforts to educate others about protective factors, do wellness activities, participate in postvention planning, and share suicide prevention resources. This finding is consistent with the literature about CoP [34], and illuminates the importance of developing a CoP to facilitate collaborative initiatives (i.e. “Working Together”) in many different locally-directed ways to reduce suicide risk and promote overall health. This clear finding suggests that creating a CoP that supports collaborative preventative activities is a promising and under-utilized avenue for a variety of strategies at multiple levels and across the prevention spectrum, especially for rural communities and those with limited institutional resources.

Shifting focus from an individual’s perceived self-efficacy to execute narrowly prescribed actions to considering their perceptions about their ability to problem solve and collaborate with others to execute locally directed actions [62] offers an expanded orientation from which to approach suicide prevention. This collective orientation is measured through our CoP independent variable and is associated with both Working Together and Postvention behaviors (see Table 4). The distinct associations include the social support systems for both those showing signs of suicide risk and for those who are supporting them. In small, tight-knit and under-resourced communities with limited professional helpers and too common suicidal events [55], this social support system for those providing support is clearly important for sustainable action.

Our findings also elucidate some important distinctions within the idea of self-efficacy for suicide prevention. Self-efficacy is a central component to many social science theories of individual behavior change. It has been incorporated into foundational implementation science frameworks, and is typically defined as “…an individual’s belief in their own abilities to execute [specific] courses of action…” [57, p. 9], This study includes two dimensions of self-efficacy: wellness and suicide prevention, signifying one’s perceived knowledge and skills to do these two types of actions. As discrete yet inter-related concepts, our Wellness Self-Efficacy and Prevention Self-Efficacy constructs are a novel way to conceptualize these two self-perceptions. Our findings corroborate previous research that self-efficacy is important, but for different kinds of behavioral outcomes. Both wellness and prevention self-efficacy are associated with offering Interpersonal Support, whereas only wellness self-efficacy is associated with Working Together. Distinctly, prevention self-efficacy is associated with Lethal Means Reduction and Postvention behaviors (see Table 4). Including both wellness promotion and suicide prevention orientations expands our understanding of the kinds of perceptions and resources needed for comprehensive prevention efforts.

These findings suggest that Regulatory Focus Theory may be an important consideration for suicide prevention, specifically the implications of promotion versus prevention framing. A wellness promotion orientation expands measurement to include commonplace events (not predicated on crisis or risk), such as listening to or offering opportunities to young people, that can promote wellbeing in an on-going way. A prevention orientation may be more aligned with stopping a rare event. While these Regulatory Focus Theory framings have been shown to impact outcomes in health communication [63, 64], and across many other domains including leadership styles [65, 66], social support [67], consumer purchasing behaviors [68], and even athletic performance [69], to our knowledge, no work has been done to investigate how Regulatory Focus constructs of prevention and promotion may be important for suicide prevention programs. Further research might explore how and for whom these dimensions—promotion and prevention—may be differently important for uptake of suicide prevention programs.

### Limitations

The study reports on cross-sectional data from remote and rural AN communities that was collected over several days in the Fall in 2019. These data were originally intended as baseline measures prior to testing the PC CARES intervention, which ultimately did not proceed in person as planned due to COVID restrictions. Under the direction of our community partners, we conducted secondary analysis of what we collected to deepen our understanding of suicide prevention processes in AN communities and to honor the effort and contributions of the AN individuals who completed the baseline surveys. Because data collection was cut short by COVID and represents a single point in time, this study cannot establish causal relationships, determine the directionality, or elucidate the temporal sequence of the associations found. Future research should be done to confirm our findings and better elucidate the causal pathways underlying the observed associations.

Although the data represents approximately 15% of the population of participating villages, the sample is not representative. We strategically recruited those in the main institutions within communities as well as people employed by institutions like the school or tribal health corporation and oversampled youth. Our survey categorized individuals according to self-reported “community” or “institutional” roles, which may not capture the nuances of how Alaska Native participants see their connections in the social environment. Indeed, 120 (30%) of our participants selected neither institutional nor community roles. This may impact our findings on role differences across our study constructs (Table 2). Additionally, the survey analysis relies on self-reported behaviors done over the last few months. Thus, our results offer just a snapshot of community members’ self-perceptions and reported suicide prevention and health promotion behaviors. It is likely that the StP survey items neglected more subtle and culturally-based forms of local support as it was limited to the survey’s prescribed behaviors. Importantly, the StP Survey measure is a work in process. Our confirmatory factor analysis (posted on Psych Archives) shows limitations of our binary (Yes/No) behavior outcomes, specifically related to our three item Lethal Means Reduction subscale. This scale has Cronbach’s alpha lower than desired. However imperfect our measure, this evidence-based construct of lethal means reduction [70–72] is a vital way to prevent suicide in communities with household firearms. We continue to work with our community partners to develop a precise and understandable subscale for this critical construct, and because of its importance, include lethal means reduction as a key behavior subscale in our analysis.

## Conclusion

Our findings overall provide foundational information for multidimensional intervention practices that support local people working collaboratively across existing institutional and community systems of care. The cross-sectional study investigates how self-perceptions related to suicide prevention, wellness and collaborative relationships are associated with suicide prevention and health promotion activities, which include behaviors across the prevention spectrum. Our study found that general support for young people (wellness or health promotion) is important to include alongside targeted suicide prevention outreach. Research suggests both types of approaches contribute to lowering youth suicide risk [73]. Given our findings, we suggest an expansion beyond focusing solely on intervening according to individual risk to include collaborations across sectors through a community of practice to encourage and support suicide prevention and wellness promotion. Moving beyond individual-level approaches to suicide prevention, this study highlights key factors in the social environment that encourage people’s engagement in activities to reduce risk and promote wellness. This expanded approach to suicide prevention is especially important for under-resourced, culturally distinct communities.

## Data Availability

The datasets generated and analyzed in the current study are not publicly available due tribal shared ownership. If interested, data can be made available from the corresponding author after going through tribal review by the Research Ethics Review Board of Norton Sound Health Corporation and Kawerak, Inc.

## References

[1] CDC. WISQARS (Web-based Injury Statistics Query and Reporting System) Injury Center [Internet]. 2021 [cited 2022 Oct 8]. Available from: https://www.cdc.gov/injury/wisqars/index.html

[2] Hedegaard H, Curtin SC, Warner M. Suicide rates in the united States continue to increase. NCHS Data Brief. 2018;(309):1–8. 30312151

[3] Martínez-Alés G, Jiang T, Keyes KM, Gradus JL. The recent rise of suicide mortality in the united States. Annu Rev Public Health. 2022;43(1):99–116. 34705474 10.1146/annurev-publhealth-051920-123206 PMC11107879

[4] Bridge JA, Ruch DA, Sheftall AH, Hahm HC, O’Keefe VM, Fontanella CA, et al. Youth suicide during the first year of the COVID-19 pandemic. Pediatrics. 2023;151(3):e2022058375. 36789551 10.1542/peds.2022-058375 PMC10227859

[5] Rural Suicide. A Systematic Review and Recommendations - Tyler R. Pritchard, Jennifer L. Buckle, Kristel Thomassin, Stephen P. Lewis, 2024 [Internet]. [cited 2025 Jan 9]. Available from: https://journals.sagepub.com/doi/full/10.1177/21677026241234319

[6] A Systematic Review of Factors Impacting Suicide Risk Among Rural Adults in the United States. - Mohatt– 2021 - The Journal of Rural Health - Wiley Online Library [Internet]. [cited 2025 Jan 9]. Available from: https://onlinelibrary.wiley.com/doi/abs/10.1111/jrh.12532 10.1111/jrh.12532 33210399

[7] David-Ferdon C, Crosby AE, Caine ED, Hindman J, Reed J, Iskander J. CDC grand rounds: preventing suicide through a comprehensive public health approach. Morb Mortal Wkly Rep. 2016;65(34):894–7. 10.15585/mmwr.mm6534a2 27584004

[8] Graves JM, Abshire DA, Mackelprang JL, Amiri S, Beck A. Association of rurality with availability of youth mental health facilities with suicide prevention services in the US. JAMA Netw Open. 2020;3(10):e2021471. 33090222 10.1001/jamanetworkopen.2020.21471 PMC7582123

[9] Alvarez K, Polanco-Roman L, Samuel Breslow A, Molock S. Structural racism and suicide prevention for ethnoracially minoritized youth: A conceptual framework and illustration across systems. Am J Psychiatry. 2022;179(6):422–33. 35599542 10.1176/appi.ajp.21101001 PMC9765395

[10] Freedenthal S, Stiffman AR. They might think I was crazy: young American Indians’ reasons for not seeking help when suicidal. J Adolesc Res. 2007;22(1):58–77.

[11] Wexler LM, Gone JP. Culturally responsive suicide prevention in Indigenous communities: unexamined assumptions and new possibilities. Am J Public Health. 2012;102(5):800–6. 22420786 10.2105/AJPH.2011.300432 PMC3483901

[12] Shields MC, Beidas RS. The need to prioritize Patient-Centered care in inpatient psychiatry as a matter of social justice. JAMA Health Forum. 2022;3(2):e214461. 36218823 10.1001/jamahealthforum.2021.4461 PMC10105342

[13] Heflinger CA, Shaw V, Higa-McMillan C, Lunn L, Brannan AM. Patterns of child mental health service delivery in a public system: rural children and the role of rural residence. J Behav Health Serv Res. 2015;42(3):292–309. 25813915 10.1007/s11414-015-9464-9

[14] Heflinger CA, Christens B. Rural behavioral health services for children and adolescents: an ecological and community psychology analysis. J Community Psychol. 2006;34(4):379–400.

[15] Howell E, McFeeters J. Children’s mental health care: differences by race/ethnicity in urban/rural areas. J Health Care Poor Underserved. 2008;19(1):237–47. 18263999 10.1353/hpu.2008.0008

[16] Murry VM, Heflinger CA, Suiter SV, Brody GH. Examining perceptions about mental health care and Help-Seeking among rural African American families of adolescents. J Youth Adolesc. 2011;40(9):1118–31. 21259067 10.1007/s10964-010-9627-1

[17] Hedegaard H, Curtin SC, Warner M. Increase in suicide mortality in the united States, 1999–2018. NCHS Data Brief. 2020;(362):1–8. 32487287

[18] Curtin SC. National vital statistics reports 69, number 11 September 11, 2020 state suicide rates among adolescents and young adults aged 10–24: United States, 2000–2018.:10. 33054915

[19] Kegler SR, Stone DM, Holland KM. Trends in suicide by level of Urbanization — United States, 1999–2015. MMWR Morb Mortal Wkly Rep. 2017;66(10):270–3. 28301448 10.15585/mmwr.mm6610a2 PMC5657870

[20] Markowski KL, White L, Harcey SR, Schmidt T, McEachern D, Habecker P et al. What kinds of support are Alaska native youth and young adults reporting?? An examination of types, quantities, sources, and frequencies of support. Health Promot Pract. 2022;152483992211150. 10.1177/15248399221115065 PMC10729876 36047453

[21] Pitman A, Caine E. The role of the high-risk approach in suicide prevention. Br J Psychiatry. 2012;201(3):175–7. 22945924 10.1192/bjp.bp.111.107805

[22] Stone DM, Simon TR, Fowler KA, Kegler SR, Yuan K, Holland KM, et al. *Vital Signs*: trends in state suicide Rates — United states, 1999–2016 and circumstances contributing to suicide — 27 states, 2015. MMWR Morb Mortal Wkly Rep. 2018;67(22):617–24. 10.15585/mmwr.mm6722a1 PMC5991813 29879094

[23] Iskander JK, Crosby AE. Implementing the National suicide prevention strategy: time for action to flatten the curve. Prev Med. 2021;152(Pt1):106734. 34344523 10.1016/j.ypmed.2021.106734 PMC8443844

[24] David-Ferdon C, Crosby AE, Caine ED, Hindman J, Reed J, Iskander J. CDC grand rounds: preventing suicide through a comprehensive public health approach. MMWR Morb Mortal Wkly Rep. 2016;65(34):894–7. 27584004 10.15585/mmwr.mm6534a2

[25] Hirsch JK, Cukrowicz KC. Suicide in rural areas: an updated review of the literature. J Rural Ment Health. 2014;38(2):65–78.

[26] Zalsman G, Hawton K, Wasserman D, van Heeringen K, Arensman E, Sarchiapone M, et al. Suicide prevention strategies revisited: 10-year systematic review. Lancet Psychiatry. 2016;3(7):646–59. 27289303 10.1016/S2215-0366(16)30030-X

[27] Steelesmith DL, Fontanella CA, Campo JV, Bridge JA, Warren KL, Root ED. Contextual factors associated with County-Level suicide rates in the united States, 1999 to 2016. JAMA Netw Open. 2019;2(9):e1910936. 31490540 10.1001/jamanetworkopen.2019.10936 PMC6735416

[28] Cramer RJ, Kapusta ND. A Social-Ecological Framework of Theory, Assessment, and Prevention of Suicide. Front Psychol [Internet]. 2017 Oct 9 [cited 2025 Jan 9];8. Available from: https://www.frontiersin.org/journals/psychology/articles/10.3389/fpsyg.2017.01756/full 10.3389/fpsyg.2017.01756 PMC5640776 29062296

[29] Standley CJ, Foster-Fishman P. Intersectionality, social support, and youth suicidality: A socioecological approach to prevention. Suicide Life Threat Behav. 2021;51(2):203–11. 33876493 10.1111/sltb.12695

[30] Chandler MJ, Lalonde C. Cultural continuity as a hedge against suicide in Canada’s first nations. Transcult Psychiatry. 1998;35(2):191–219.

[31] O’Keefe VM, Maudrie TL, Cole AB, Ullrich JS, Fish J, Hill KX, et al. Conceptualizing Indigenous strengths-based health and wellness research using group concept mapping. Arch Public Health. 2023;81(1):71. 37101194 10.1186/s13690-023-01066-7 PMC10134608

[32] Kirmayer LJ, Gone JP, Moses J. Rethinking Hist Trauma Transcult Psychiatry. 2014;51(3):299–319. 10.1177/1363461514536358 24855142

[33] Pham TV, Fetter AK, Wiglesworth A, Rey LF, Prairie Chicken ML, Azarani M, et al. Suicide interventions for American Indian and Alaska native populations: A systematic review of prevention strategies, logics, and rationales. SSM - Ment Health. 2022;2:100139.

[34] Wenger E, McDermott RA, Snyder W. Cultivating communities of practice: a guide to managing knowledge. Boston: Harvard Business School Press; 2002.

[35] Trout L, McEachern D, Mullany A, White L, Wexler L. Decoloniality as a framework for Indigenous youth suicide prevention pedagogy: promoting community conversations about research to end suicide. Am J Community Psychol. 2018;62(3–4):396–405. 30561803 10.1002/ajcp.12293 PMC6300065

[36] Wenger E. Communities of practice: learning, meaning, and identity. Cambridge University Press; 1999.

[37] Li LC, Grimshaw JM, Nielsen C, Judd M, Coyte PC, Graham ID. Evolution of Wenger’s concept of community of practice. Implement Sci. 2009;4(1):11. 19250556 10.1186/1748-5908-4-11 PMC2654669

[38] Wexler L, White LA, O’Keefe VM, Rasmus S, Haroz EE, Cwik MF, et al. Centering community strengths and resisting structural racism to prevent youth suicide: learning from American Indian and Alaska native communities. Arch Suicide Res. 2024;0(0):1–16. 10.1080/13811118.2023.2300321 PMC11258209 38240632

[39] Reid P, Cormack D, Paine SJ. Colonial histories, racism and health—The experience of Māori and Indigenous peoples. Public Health. 2019;172:119–24. 31171363 10.1016/j.puhe.2019.03.027

[40] Wexler L, McEachern D, DiFulvio G, Smith C, Graham LF, Dombrowski K. Creating a community of practice to prevent suicide through multiple channels: describing the theoretical foundations and structured learning of PC CARES. Int Q Community Health Educ. 2016;36(2):115–22. 26880738 10.1177/0272684X16630886 PMC4794395

[41] Trout L, Wexler L, Arctic, Suicide. Social medicine, and the purview of care in global mental health. Health Hum Rights. 2020;22(1):77–89. PMC7348442 32669791

[42] Holmes G, Clacy A, Hermens DF, Lagopoulos J. The Long-Term efficacy of suicide prevention gatekeeper training: A systematic review. Arch Suicide Res. 2021;25(2):177–207. 31809659 10.1080/13811118.2019.1690608

[43] Cesario J, Higgins ET, Scholer AA. Regulatory fit and persuasion: basic principles and remaining questions. Soc Personal Psychol Compass. 2008;2(1):444–63.

[44] Higgins ET. Beyond pleasure and pain. Am Psychol. 1997;52(12):1280–300. 9414606 10.1037//0003-066x.52.12.1280

[45] Michelmore L, Hindley P. Help-Seeking for suicidal thoughts and Self-Harm in young people: A systematic review. Suicide Life Threat Behav. 2012;42(5):507–24. 22889130 10.1111/j.1943-278X.2012.00108.x

[46] Wexler L, Trout L, Rataj S, Kirk T, Moto R, McEachern D. Promoting community conversations about research to end suicide: learning and behavioural outcomes of a training-of-trainers model to facilitate grassroots community health education to address Indigenous youth suicide prevention. Int J Circumpolar Health. 2017;76(1):1345277. 28762305 10.1080/22423982.2017.1345277 PMC5549821

[47] Wexler L, Rataj S, Ivanich J, Plavin J, Mullany A, Moto R, et al. Community mobilization for rural suicide prevention: process, learning and behavioral outcomes from promoting community conversations about research to end suicide (PC CARES) in Northwest Alaska. Soc Sci Med 1982. 2019;232:398–407. 10.1016/j.socscimed.2019.05.028 PMC6925945 31151026

[48] White LA, Wexler L, Weaver A, Moto R, Kirk T, Rataj S, et al. Implementation beyond the clinic: Community-driven utilization of research evidence from PC CARES, a suicide prevention program. Am J Community Psychol. 2022;70(3–4):365–78. 35762450 10.1002/ajcp.12609 PMC10084270

[49] Wexler L, Schmidt T, White L, Wells CC, Rataj S, Moto R, et al. Collaboratively adapting Culturally-Respectful, Locally-Relevant suicide prevention for newly participating Alaska native communities. J Soc Action Couns Psychol. 2022;14(1):124–51.

[50] Wells CC, White L, Schmidt T, Rataj S, McEachern D, Wisnieski D, et al. Adapting PC CARES to continue suicide prevention in rural Alaska during the COVID-19 pandemic: narrative overview of an In-Person Community-Based suicide prevention program moving online. Am Indian Alsk Native Ment Health Res. 2022;29(2):126–54. 35881985 10.5820/aian.2902.2022.126 PMC10732495

[51] Kapoulea E, Ginn J, White L, Schmidt T, Rataj S, Habecker P et al. Developing the Steps to Prevention Scale: Confirmatory Factor Analysis of the Promoting Community Conversations About Research to End Suicide (PC CARES) Study Measures [Internet]. PsychArchives; 2023. Available from: Link available on request.

[52] IBM Corp. IBM SPSS Statistics for Windows. 2020.

[53] Holm S. A simple sequential rejective multiple test procedure. Scand J Stat. 1979;6:65–70.

[54] Leavitt RA, Ertl A, Sheats K, Petrosky E, Ivey-Stephenson A, Fowler KA. Suicides among American Indian/Alaska Natives — National violent death reporting system, 18 States, 2003–2014. MMWR Morb Mortal Wkly Rep. 2018;67(8):237–42. 29494572 10.15585/mmwr.mm6708a1 PMC5861703

[55] Wexler L, Silveira ML, Bertone-Johnson E. Factors associated with Alaska native fatal and nonfatal suicidal behaviors 2001–2009: trends and implications for prevention. Arch Suicide Res. 2012;16(4):273–86. 23137218 10.1080/13811118.2013.722051

[56] Burnette C, Ramchand R, Ayer L. Gatekeeper training for suicide prevention. Rand Health Q. 2015;5(1):16. 28083369 10.7249/rhq-05-01-16 PMC5158249

[57] Pistone I, Beckman U, Eriksson E, Lagerlöf H, Sager M. The effects of educational interventions on suicide: A systematic review and meta-analysis. Int J Soc Psychiatry. 2019;65(5):399–412. 31159627 10.1177/0020764019852655

[58] Morton M, Wang S, Tse K, Chung C, Bergmans Y, Ceniti A, et al. Gatekeeper training for friends and family of individuals at risk of suicide: A systematic review. J Community Psychol. 2021;49(6):1838–71. 34125969 10.1002/jcop.22624

[59] Robinson-Link N, Hoover S, Bernstein L, Lever N, Maton K, Wilcox H. Is gatekeeper training enough for suicide prevention?? School Ment Health. 2020;12(2):239–49.

[60] Grattidge L, Hoang H, Mond J, Lees D, Visentin D, Auckland S. Exploring Community-Based suicide prevention in the context of rural Australia: A qualitative study. Int J Environ Res Public Health. 2023;20(3):2644. 10.3390/ijerph20032644. PMID: 36768008; PMCID: PMC9915251. 36768008 10.3390/ijerph20032644 PMC9915251

[61] Meza JI, Bath E. One size does not fit all: making suicide prevention and interventions equitable for our increasingly diverse communities. J Am Acad Child Adolesc Psychiatry. 2021;60(2):209–12. 33068754 10.1016/j.jaac.2020.09.019

[62] White LA, Wexler L, Weaver A, Moto R, Kirk T, Rataj S et al. Implementation beyond the clinic: Community-driven utilization of research evidence from PC CARES, a suicide prevention program. Am J Community Psychol. 2022. 10.1002/ajcp.12609 PMC10084270 35762450

[63] Fridman I, Higgins ET. Regulatory Focus and Regulatory Fit in Health Messaging. In: Oxford Research Encyclopedia of Communication [Internet]. 2017 [cited 2025 Jan 21]. Available from: https://oxfordre.com/communication/display/10.1093/acrefore/9780190228613.001.0001/acrefore-9780190228613-e-257?d=%2F10.1093%2Facrefore%2F9780190228613.001.0001%2Facrefore-9780190228613-e-257%26p=emailAWljOf7PqiQeM

[64] Ludolph R, Schulz PJ. Does regulatory fit lead to more effective health communication? A systematic review. Soc Sci Med. 2015;128:142–50. 25617673 10.1016/j.socscimed.2015.01.021

[65] Johnson PD, Smith MB, Wallace JC, Hill AD, Baron RA. A review of multilevel regulatory focus in organizations. J Manag. 2015;41(5):1501–29.

[66] Kark R, Van Dijk D. Motivation to lead, motivation to follow: the role of the Self-Regulatory focus in leadership processes. Acad Manage Rev. 2007;32(2):500–28.

[67] Giving the Help That Is Needed: How Regulatory Mode Impacts Social Support -, Cavallo JV, Zee KS. E. Tory Higgins, 2016 [Internet]. [cited 2025 Jan 21]. Available from: https://journals.sagepub.com/doi/abs/10.1177/0146167216651852 10.1177/0146167216651852 27354111

[68] Werth L, Foerster J. How regulatory focus influences consumer behavior. Eur J Soc Psychol. 2007;37(1):33–51.

[69] Plessner H, Unkelbach C, Memmert D, Baltes A, Kolb A. Regulatory fit as a determinant of sport performance: how to succeed in a soccer penalty-shooting. Psychol Sport Exerc. 2009;10(1):108–15.

[70] Mann JJ, Apter A, Bertolote J, Beautrais A, Currier D, Haas A, et al. Suicide prevention strategies: A systematic review. JAMA. 2005;294(16):2064–74. 16249421 10.1001/jama.294.16.2064

[71] Mann JJ, Michel CA, Auerbach RP. Improving suicide prevention through Evidence-Based strategies: A systematic review. Am J Psychiatry. 2021;178(7):611–24. 33596680 10.1176/appi.ajp.2020.20060864 PMC9092896

[72] Yip PS, Prof CE, Prof, Yousuf SFCPS, Chang SS, PhD. Wu KCC phd, Chen YY dr. Means restriction for suicide prevention. Lancet Br Ed. 2012;379(9834):2393–9. 10.1016/S0140-6736(12)60521-2 PMC6191653 22726520

[73] Shahram SZ, Smith ML, Ben-David S, Feddersen M, Kemp TE, Plamondon K. Promoting zest for life: A systematic literature review of resiliency factors to prevent youth suicide. J Res Adolesc. 2021;31(1):4–24. 33665921 10.1111/jora.12588 PMC7986824

